# Spontaneous Atypical Meningioma in a Pregnant Rhesus Macaque ( *Macaca mulatta* )

**DOI:** 10.1111/jmp.70049

**Published:** 2025-12-01

**Authors:** Eddie Xu, Avelina Rodgers, Katherine Johnson, Robert V. Blair

**Affiliations:** ^1^ Tulane National Biomedical Research Center Covington Louisiana USA

## Abstract

Spontaneous meningiomas are rarely reported in nonhuman primates, and among the documented cases, only a few are thoroughly characterized. This report describes a case of atypical meningioma in a pregnant rhesus macaque ( *Macaca mulatta* ) that exhibits pathobiological features that mirror humans.

## 1 Introduction

In humans, brain and other central nervous system (CNS) tumors account for an estimated 24 000 new cases every year and are notorious for their high mortality rates compared to other cancers [1, 2]. Their clinical manifestations are highly variable and often depend on the tumor's location and rate of growth [3]. The development of therapeutic strategies has proven difficult due to the lack of models that faithfully recapitulate the pathobiology of human brain tumors. This report aims to compare the pathobiological features of a high‐grade meningioma between species and to highlight potential hormonal influences.

## 2 Case Report

All procedures adhere to the ethical policies of the journal. Ethical approval was not required as this was a spontaneous case, and the animal in this case report was not enrolled in a study.

A 5.93‐year‐old pregnant female rhesus macaque ( *Macaca mulatta* ) weighing 6.77 kg that was born and raised at the Tulane National Biomedical Research Center (Covington, LA, USA) presented during its second pregnancy due to neurological signs. At GD ~141, a physical examination found the pupils to be dilated bilaterally and the animal exhibiting a decreased response to stimuli. Complete blood count returned all values within reference ranges, and clinical chemistry revealed mildly elevated potassium (4.7 mEq/L, RR: 3.4–4.3), globulin (3.7 g/dL, RR: 2.0–3.3), and BUN (37.5 mg/dL, RR: 12.3–24.8). Cerebrospinal fluid (CSF) analysis revealed no evidence of pleocytosis when corrected for peripheral blood contamination. Given the lack of an inflammatory leukogram or CSF pleocytosis, the animal was started on sustained release buprenorphine and frequent observations. At the follow‐up exam the next day, the animal appeared depressed with persistent mydriasis and absent pupillary light reflexes bilaterally. No other ocular abnormalities were noted. The animal progressed to obtundation and was sent for humane euthanasia before further therapy could be initiated.

**FIGURE 1:**
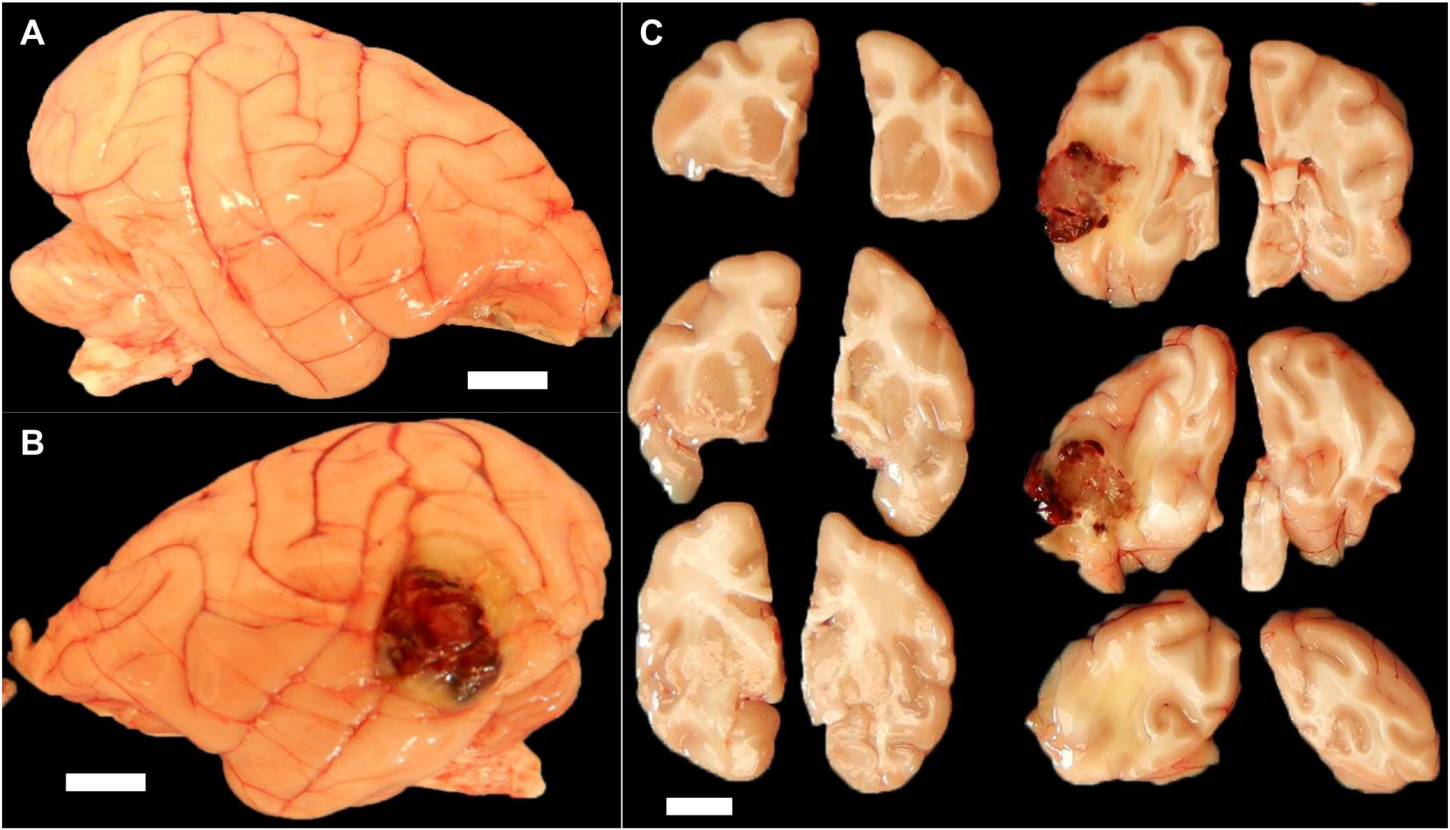
Gross pathology of a brain tumor in a rhesus macaque. (A) Right hemisphere normal gross appearance. (B) There is a 2 × 2 × 2 cm red to black mass in the left temporal lobe. (C) On coronal section, the mass is partially circumscribed and exhibits local invasion. Bar = 1 cm.

**FIGURE 2:**
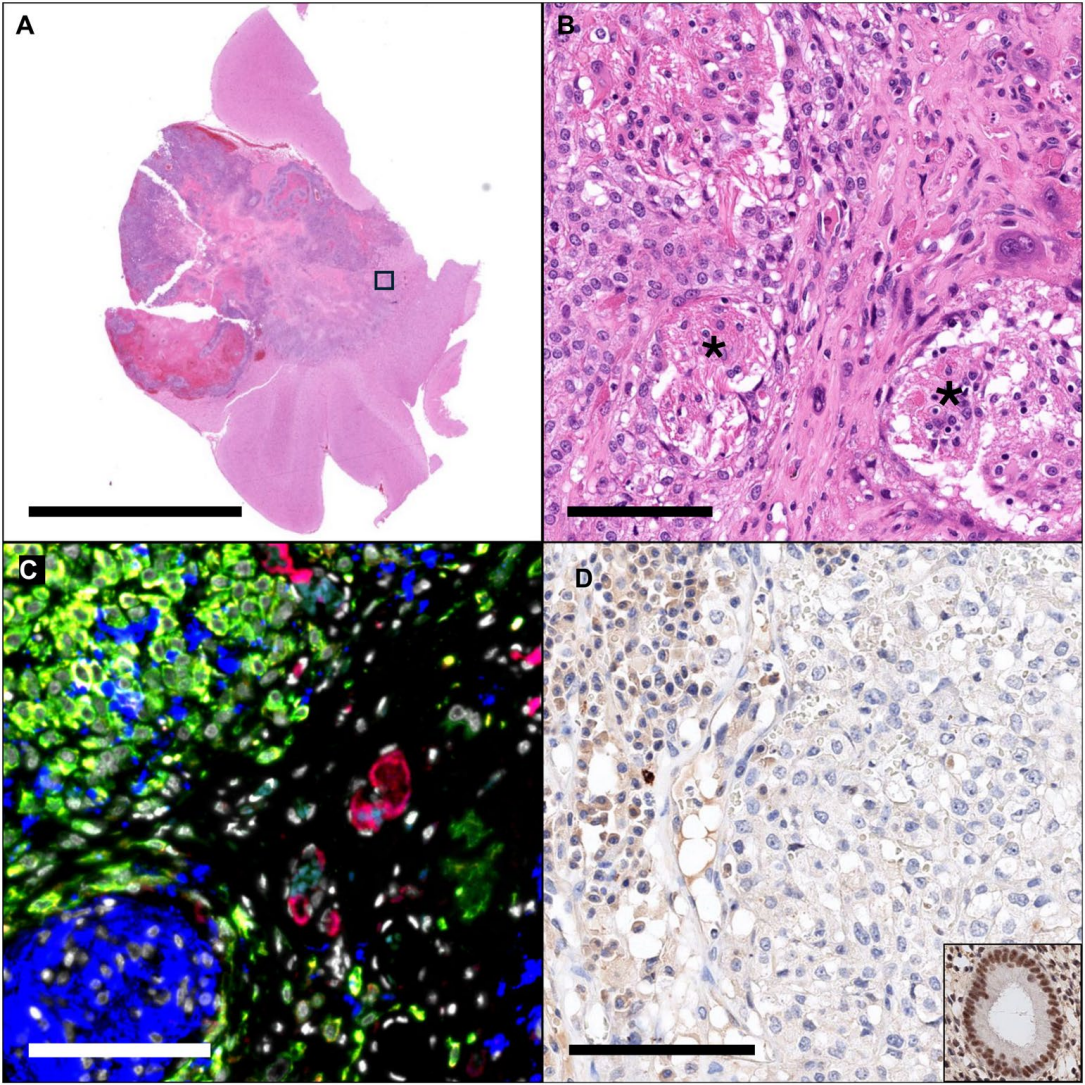
Histopathologic and immunohistochemical characterization of the neoplasm. (A) Subgross image of the mass, invading, expanding and effacing the temporal lobe. Bar = 1 cm. (B) (boxed region in A). Neoplastic cells are polygonal, with oval nuclei, finely stippled chromatin, and scant, finely granular eosinophilic cytoplasm with indistinct cell borders. Neoplastic cells occasionally invade and surround bundles of white matter (asterisks). Bar = 100 um. (C) Neoplastic cells exhibit diffuse cytoplasmic expression of CK (green) and EMA (red, yellow when colocalized) and are negative for GFAP (blue) Bar = 100 cm. (D) Neoplastic cells (right) are negative for progesterone receptor (brown). Inset: Positive control for progesterone receptor in an endometrial gland. Bar = 100. DAB.

Postmortem examination identified a heterogeneous, 2 cm diameter, red to black, slightly firm mass located within the left temporal lobe (Figure 1). On the cut surface, the mass invaded into deep brain structures (Figures 1A–C). No other significant gross abnormalities were observed. Microscopic examination of the left temporal lobe mass revealed a densely cellular, unencapsulated neoplasm invading the temporal cortex and subjacent white matter (Figure 2A). The neoplasm was composed of sheets of polygonal cells supported on a scant fibrovascular stroma. There were multifocal to coalescing areas of hemorrhage and necrosis throughout the neoplasm that comprised 20%–30% of the neoplasm. Neoplastic cells had round to oval nuclei with finely stippled chromatin and 1–3 prominent nucleoli. Neoplastic cells had scant, finely granular, eosinophilic to clear cytoplasm with indistinct cell borders. Neoplastic cells exhibited moderate to marked anisocytosis and anisokaryosis. The mitotic index was 15 mitoses in 10 high‐powered fields. The neoplasm exhibited extensive local invasion, often surrounding and isolating bundles of white matter along its margin (Figure 2B). Immunohistochemistry revealed strong and diffuse cytoplasmic expression of cytokeratin (CK) and epithelial membrane antigen (EMA) in neoplastic cells (Figure 2C). Neoplastic cells were diffusely negative for GFAP and progesterone receptor (Figure 2D).

Taken together, the pathological and immunohistochemical findings are consistent with a grade II atypical meningioma.

## 3 Discussion

In humans, meningiomas are the most common type of intracranial tumor. The majority of meningiomas are grade I, mostly benign, and characterized by slow growth. In contrast, atypical meningiomas are grade II tumors that exhibit local invasion, higher mitotic rates, and have greater recurrence rates. Although less aggressive than grade III malignant meningiomas, they display histological and clinical features that fall between grades I and III [4, 5, 6, 7, 8]. Clinical signs vary depending on location but often include focal neurological deficits due to compression of adjacent brain regions, as was seen in the case described above. To our knowledge, only seven cases of spontaneous occurring meningiomas have been reported in nonhuman primates [8, 9, 10, 11, 12, 13, 14]. Among the seven reported cases, only one other case was reported in a rhesus macaque [8]. For the five nonhuman primate cases in which sex information was available, four out of five were female. Although the predominance of cases in females may be influenced by breeding colony demographics which typically maintain a higher proportion of females, it is notable that our case was female as well. This mirrors the prevalence in humans wherein meningiomas are more common in women [8, 15, 16, 17, 18, 19]. This sex disparity is likely attributable to differences in progesterone levels between males and females, as progesterone has been implicated in meningioma growth [20, 21]. This association is further supported by clinical observations in individuals receiving synthetic progestins; meningiomas may grow and become symptomatic, and subsequently stabilize or regress following discontinuation of the hormone [20]. Pregnancy presents a unique risk due to the progressive rise in progesterone levels throughout gestation. Notably, the rhesus macaque in our study was late in her third trimester of pregnancy, and therefore, it is possible that endogenous hormones contributed to the progression of her clinical signs. In humans, there are multiple reports of meningiomas during pregnancy. These cases demonstrate how meningiomas respond to hormonal changes, initially with growth, followed by stabilization or regression after delivery [16, 21, 22]. Interestingly, expression of progesterone receptors is inversely correlated with favorable prognoses [23, 24]. Grade I meningiomas exhibit the highest levels of progesterone receptor expression, whereas grades II and III demonstrate progressively lower expression levels. The neoplasm in our case was diffusely negative for progesterone receptor expression, consistent with its higher grade, malignant behavior, and poor outcome. Additionally, the strong CK expression further supports this as a high‐grade meningioma. CK expression is more common in higher grade meningiomas and rare in benign meningiomas, with the exception of secretory meningiomas [25, 26]. The meningioma in our case was an atypical meningioma characterized by its patternless growth, regions of necrosis, and mitotic rate of 15 mitoses in 10 high‐powered fields. To our knowledge, this is the first case of an atypical meningioma described in a pregnant nonhuman primate. This case report describes the comparative pathology of meningioma in humans and nonhuman primates and highlights shared pathobiology that drives similar sex disparities and complications during pregnancy.

## Funding

This work was supported by the National Institutes of Health, P51OD011104.

## Conflicts of Interest

The authors declare no conflicts of interest.

## Data Availability

The data that support the findings of this study are openly available in Figshare at https://figshare.com/s/cb99a4e6285fa12bd07e.
